# Integrated multi-omics, network pharmacology, and experimental validation reveal the protective mechanisms of curcumol against DSS-induced ulcerative colitis

**DOI:** 10.3389/fimmu.2026.1852138

**Published:** 2026-08-28

**Authors:** Fu Shu, Kaiqi Chen, Yingjie Song, Jiahao Li, Mujun Shen, Zhiwei Guo, Hongyan Lai, Yue Xu, Na Lei, Fajuan Zheng, Zhen Zhang

**Affiliations:** 1 College of Pharmaceutical Sciences, Southwest University, Chongqing, China; 2 Department of Anorectal Surgery, Chongqing Hospital of Traditional Chinese Medicine, Chongqing, China; 3 Science and Technology & Foreign Affairs Division, Chongqing Hospital of Traditional Chinese Medicine, Chongqing, China

**Keywords:** Curcumol, gut microbiota, metabolomics, transcriptomics, ulcerative colitis

## Abstract

**Background:**

Ulcerative colitis (UC) is a chronic relapsing inflammatory bowel disease characterized by mucosal immune imbalance, epithelial barrier disruption, and intestinal dysbiosis. Curcumol (CUR) possesses anti-inflammatory and immunomodulatory activities; however, the multilevel mechanisms underlying its protective effects against dextran sulfate sodium (DSS)-induced UC remain incompletely understood.

**Methods:**

A mouse model of UC was established in C57BL/6J mice using 3% DSS. CUR or 5-aminosalicylic acid (5-ASA) was administered once daily beginning on the first day of DSS exposure, with 5-ASA serving as a phenotypic positive control. The protective effects of CUR were evaluated based on body weight change, disease activity index (DAI), colon length, histopathological alterations, inflammatory cytokine levels, and barrier-related molecules. Based on the pharmacodynamic and phenotypic evaluation, the high-dose CUR group was selected for subsequent mechanistic studies, including fecal 16S rRNA gene sequencing, fecal untargeted metabolomics, colonic transcriptome sequencing, analysis of public human UC transcriptomic datasets, and integrative network pharmacology, followed by experimental validation of key candidate molecules.

**Results:**

CUR significantly ameliorated DSS-induced body weight loss, reduced DAI scores, preserved colon length, and alleviated histopathological injury. CUR also decreased the levels of TNF-α, IL-1β, IL-6, and IL-17, restored goblet cell abundance, and partially restored the protein expression of MUC2, claudin-1, ZO-1, and occludin. Moreover, these protective effects were generally more pronounced in the high-dose CUR group, with significant between-dose differences observed for selected endpoints (P < 0.05). Further exploratory analyses showed that high-dose CUR partially restored gut microbial diversity and reshaped community structure while partially reversing DSS-induced metabolic disturbances. Integrative analysis of mouse colonic transcriptomics, public human UC transcriptomic datasets, and network pharmacology converged on SPP1 and CD44 as candidate hub genes and implicated the PI3K/Akt signaling pathway as a key candidate pathway altered by DSS exposure and modulated by CUR. Experimental validation further demonstrated that high-dose CUR reduced the expression of SPP1, CD44, and PIK3CB and decreased the p-p85/p85 and p-Akt/Akt ratios. Multiplex immunofluorescence further showed a reduction in the macrophage-associated fraction of SPP1-positive cells after high-dose CUR intervention.

**Conclusions:**

Concurrent administration of CUR exerted protective effects in a DSS-induced mouse model of UC by reducing inflammation and mucosal barrier injury and partially normalizing gut microbial and fecal metabolic profiles. These effects were associated with attenuation of SPP1/CD44/PI3K/Akt-related signaling, suggesting that this signaling network may contribute to CUR-mediated protection against DSS-induced UC.

## 1 Introduction

Ulcerative colitis (UC) is one of the major clinical subtypes of inflammatory bowel disease (IBD) and is characterized by chronic relapsing inflammation, a prolonged disease course, and marked clinical heterogeneity, all of which can continuously impair patients’ quality of life and impose a substantial social burden (1–3). Although 5-aminosalicylic acid (5-ASA), glucocorticoids, immunosuppressants, and biologics are widely used in UC therapy, a considerable proportion of patients still experience inadequate therapeutic response, difficulty maintaining remission, concerns about the safety of long-term medication, and a heavy economic burden (3). Therefore, the development of naturally derived bioactive small molecules with multi-target regulatory potential and favorable safety profiles remains an important direction in basic and translational UC research.

UC is currently regarded as a disease not driven by a single pathological event, but by long-term interactions among mucosal immune dysregulation with amplified inflammatory responses, epithelial barrier damage, and intestinal microecological imbalance (4–7). In this process, disruption of the mucus layer and tight junctions increases intestinal permeability, allowing luminal antigens, bacteria, and their products to translocate more readily and further amplify lamina propria inflammation. Meanwhile, microbe-derived metabolites can reshape mucosal immune status and epithelial repair capacity through short-chain fatty acid, tryptophan, bile acid, and lipid metabolic pathways, thereby forming a pathological network in which inflammation, barrier dysfunction, and microecology/metabolism reinforce one another (5–7).

Curcumae Rhizoma is a traditional medicinal plant rich in volatile oils and sesquiterpenoids, and previous studies have suggested that it can modulate UC-related inflammation and barrier abnormalities (8). Curcumol (CUR), an important sesquiterpene constituent of Curcumae Rhizoma, possesses anti-inflammatory, immunomodulatory, hepatoprotective, and antitumor activities (9). Pharmacokinetic studies have shown that orally administered CUR exhibits relatively high tissue exposure in the small intestine and colon (10). A recent study further showed that CUR ameliorates dextran sulfate sodium (DSS)-induced UC by activating pregnane X receptor (PXR) and transcriptionally repressing stimulator of interferon genes (STING) signaling (11). However, existing studies have mainly focused on single inflammatory signaling axes, and it remains unclear whether CUR can simultaneously improve inflammatory responses, mucosal barrier injury, and gut microbiota–metabolic dysregulation, as well as which key host-side molecular mechanisms may be involved.

Integrated multi-omics approaches can simultaneously characterize changes in host transcription, microbial composition, and metabolic profiles, and are therefore well suited for dissecting complex diseases such as UC that involve multilevel interactions (12–14). Based on this rationale, the present study used a DSS-induced UC mouse model and systematically evaluated the pharmacodynamic phenotypes, inflammatory responses, mucosal barrier, and microbiota–metabolism homeostasis associated with CUR using 16S rRNA sequencing, fecal untargeted metabolomics, and multiple molecular biological assays. Combined with mouse colonic transcriptomics, mining of public human UC transcriptomic datasets, and integrative network pharmacology analysis, we further screened for key candidate molecules and signaling pathways potentially involved in the effects of CUR.

## 2 Methods

### 2.1 Animals

Specific pathogen-free (SPF) male C57BL/6J mice, 6–8 weeks old and weighing approximately 20 g, were purchased from Beijing HFK Bioscience Co., Ltd. All mice were acclimatized for 7 days under constant temperature (22 ± 2 °C) and a 12 h/12 h light/dark cycle, with free access to food and water.

### 2.2 DSS-induced UC model establishment and drug administration

After acclimatization, mice were stratified by baseline body weight and randomly allocated within strata to five groups (n = 12 per group): control (CON), DSS only (DSS), DSS plus 5-ASA (100 mg/kg/day), DSS plus low-dose CUR (CUR-L; 12.5 mg/kg/day), and DSS plus high-dose CUR (CUR-H; 50 mg/kg/day). CUR (CAS No. 4871-97-0; catalog No. HY-N0104; purity, 99.5%) was purchased from MedChemExpress (Monmouth Junction, NJ, USA). All groups except the CON group received 3% (w/v) DSS in drinking water ad libitum for 7 consecutive days to induce UC, whereas CON mice received distilled water ad libitum. During the same 7-day period, 5-ASA or CUR was administered once daily by oral gavage at the indicated dose, whereas mice in the CON and DSS groups received an equivalent volume of vehicle. The 5-ASA dose was selected based on a previously reported DSS-colitis protocol using 100 mg/kg/day by oral gavage (15). The CUR-H dose was selected on the basis of a previous *in vivo* dose-ranging study in which curcumol was administered orally at 12.5–50 mg/kg/day and pharmacokinetic data demonstrating high small-intestinal and colonic exposure after oral administration (10, 16), whereas CUR-L was included as an exploratory dose to assess dose-related effects. Clinical status, body weight, stool consistency, and fecal bleeding were assessed daily. The disease activity index (DAI) was calculated daily from body-weight loss, stool consistency, and fecal bleeding according to a previously described scoring system (15). For an exploratory assessment of hepatic and renal biochemical safety at the high CUR dose, serum alanine aminotransferase (ALT) and aspartate aminotransferase (AST) activities and serum creatinine and urea concentrations were measured in a subset of mice from the CON, DSS, and CUR-H groups (n = 6 per group) using an automated chemistry analyzer (BS-460; Shenzhen Mindray Bio-Medical Electronics Co., Ltd., Shenzhen, China) in accordance with the manufacturer’s instructions.

### 2.3 Pharmacodynamic evaluation, sample collection, and selection of samples for subsequent omics analyses

At the end of the experiment, mice were deeply anesthetized with 3% isoflurane inhalation and euthanized by cervical dislocation under deep anesthesia. Death was confirmed before tissue collection. The colon was then isolated and measured. Distal colon tissues were fixed in 4% paraformaldehyde for histopathology, immunohistochemistry (IHC), and immunofluorescence (IF); the remaining colonic tissues were snap-frozen in liquid nitrogen for reverse transcription quantitative polymerase chain reaction (RT-qPCR), Western blotting, and RNA sequencing (RNA-seq). Fresh fecal samples were used for 16S rRNA gene sequencing and untargeted metabolomics, and serum was collected for enzyme-linked immunosorbent assay (ELISA). For exploratory omics profiling, the CON, DSS, and CUR-H groups were selected on the basis of group-level pharmacodynamic and phenotypic outcomes. Four mice per group were selected, and samples from individual mice were treated as independent biological replicates. Fecal 16S rRNA gene sequencing, fecal untargeted metabolomics, and colonic RNA-seq were therefore performed with four biological replicates per group. Omics profiling was not performed in the CUR-L or 5-ASA groups; accordingly, omics-based comparisons were restricted to the CON, DSS, and CUR-H groups. Unless otherwise specified, “CUR” in the omics results below refers to the CUR-H group.

### 2.4 Hematoxylin and eosin and periodic acid–Schiff staining

Colon tissues were paraffin-embedded and sectioned (4 μm). Hematoxylin and eosin (H&E) staining was used to evaluate histological changes, and pathological scoring was performed according to previous reports (13, 15). Periodic acid–Schiff (PAS) staining was used to assess changes in goblet cell numbers. Before evaluation, all sections were labeled with coded sample numbers. H&E histopathological scoring and manual counting of PAS-positive goblet cells were independently performed by two investigators who were blinded to group allocation. For each mouse, three non-overlapping fields were evaluated at 150× magnification using the same field-selection rules, and field-level values were averaged to obtain one biological value for statistical analysis.

### 2.5 ELISA

According to the manufacturers’ instructions, levels of TNF-α, IL-1β, IL-6, and IL-17 were measured in serum and colonic tissue homogenates, and the serum concentration of osteopontin (OPN; encoded by *SPP1*) was also measured. Information on ELISA kits is provided in **Supplementary Table 1**.

### 2.6 RT-qPCR

Total RNA from colonic tissues was extracted using TRIzol and reverse-transcribed, followed by RT-qPCR using a SYBR Green system. *Gapdh* was used as the internal control gene, and relative expression levels of target genes were calculated using the 2^-ΔΔCt^ method. The genes assayed included *Muc2*, *Cldn1*, *Tjp1*, and *Ocln*. Primer sequences are listed in **Supplementary Table 2**.

### 2.7 Immunohistochemistry

The IHC procedure was based on methods previously published by our group and was optimized according to the antibody conditions used in the present study (17, 18). After deparaffinization, rehydration, antigen retrieval, and blocking, paraffin sections were incubated with primary antibodies overnight at 4 °C. Sections were then incubated with horseradish peroxidase (HRP)-conjugated secondary antibodies and visualized with 3,3′-diaminobenzidine (DAB). After hematoxylin counterstaining, images were acquired under identical microscope settings. Marker-positive area was calculated using a uniform threshold as positive area divided by the analyzed region of interest; three non-overlapping fields were averaged to obtain one biological value per mouse. Antibody information for IHC, IF, and Western blotting is summarized in **Supplementary Table 3**.

### 2.8 Immunofluorescence

Conventional IF was performed according to previously published methods from our group (17, 18). After deparaffinization, rehydration, antigen retrieval, and blocking, sections were incubated with primary antibodies overnight at 4 °C, followed by corresponding fluorescent secondary antibodies and 4′,6-diamidino-2-phenylindole (DAPI) nuclear counterstaining. Images were acquired under identical exposure conditions, and marker-positive area fractions were quantified using uniform thresholds. To assess the cellular localization of OPN/SPP1, multiplex tyramide signal amplification (TSA) IF was performed using a three-marker/four-color kit (Cat. No. PK10032; Wuhan Sanying Biotechnology, Inc., Wuhan, China). OPN/SPP1, F4/80, and pan-cytokeratin (Pan-CK) were detected sequentially through repeated cycles of primary-antibody incubation, HRP-mediated tyramide deposition, and antibody stripping, followed by DAPI counterstaining. SPP1-positive cells were expressed as a percentage of all DAPI-positive cells. To characterize the cellular distribution of the SPP1-positive population, the percentages of SPP1-positive cells co-expressing F4/80 or Pan-CK were calculated separately using all SPP1-positive cells as the denominator. Three non-overlapping fields were averaged to obtain one biological value per mouse.

### 2.9 Western blotting

Total protein was extracted from colonic tissues using radioimmunoprecipitation assay (RIPA) lysis buffer and quantified by the bicinchoninic acid (BCA) method. Equal amounts of protein were separated by sodium dodecyl sulfate–polyacrylamide gel electrophoresis (SDS-PAGE) and transferred to polyvinylidene fluoride (PVDF) membranes. After blocking, membranes were incubated with primary antibodies and HRP-conjugated secondary antibodies, and signals were detected by enhanced chemiluminescence (ECL). Densitometric values were analyzed using ImageJ. SPP1, CD44, and PI3K p110β (encoded by PIK3CB) were normalized to GAPDH; phospho-p85 and phospho-Akt were normalized to total p85 and Akt, respectively. Antibodies are listed in **Supplementary Table 3**.

### 2.10 Fecal 16S rRNA sequencing and bioinformatics analysis

Four fecal samples from each of the CON, DSS, and CUR-H groups were used for 16S rRNA sequencing. Sample preprocessing, total DNA extraction, PCR amplification, library construction, and sequencing were performed by Personal Biotechnology Co., Ltd. (Shanghai, China). Briefly, 0.2–0.5 g of fecal sample was homogenized in extraction lysis buffer using a Tissuelyser-48 tissue grinder. Total microbial DNA was extracted using the Mag-Bind Soil DNA Kit (Omega Bio-tek, Norcross, GA, USA), and DNA quality and concentration were assessed by 0.8% agarose gel electrophoresis and NanoDrop spectrophotometry. The V3–V4 region of the bacterial 16S rRNA gene was then amplified using the 338F/806R primers, and the libraries were sequenced on an Illumina NovaSeq 6000 platform with 2 × 250 bp paired-end reads. Raw sequences were processed in QIIME 2 (version 2019.4) using the demux plugin for demultiplexing, primer trimming with cutadapt, and denoising, merging, and chimera removal using DADA2 to generate an amplicon sequence variant (ASV) table (19, 20). Rare ASVs with abundance below 0.001% of the total sequencing depth across all samples were removed, and the denoised ASV matrix was used for subsequent analyses. All fecal samples were processed, used for library construction, sequenced, and analyzed within the same project workflow and sequencing batch; therefore, no separate sequencing-batch correction was required. After rarefaction to 95% of the minimum sequencing depth among all samples, α-diversity indices including Good’s coverage, Chao1, Shannon, and Faith’s PD were calculated. This rarefaction criterion was also used by the built-in α- and β-diversity procedures to minimize sequencing-depth bias. Principal coordinates analysis (PCoA), uniform manifold approximation and projection (UMAP), and non-metric multidimensional scaling (NMDS) were performed based on distance matrices, and permutational multivariate analysis of variance (PERMANOVA) was used to evaluate intergroup differences in community structure. Linear discriminant analysis effect size (LEfSe; LDA score > 4) was used to identify differential taxa, and PICRUSt2 was used to predict potential functional pathways (21). In addition, the normalized stochasticity ratio (NST) and the neutral community model were applied descriptively to assess the relative contributions of stochastic and deterministic processes to community assembly.

### 2.11 Fecal untargeted metabolomics analysis

Four fecal samples from each of the CON, DSS, and CUR-H groups were subjected to fecal untargeted metabolomics. Sample extraction, liquid chromatography–mass spectrometry (LC-MS) analysis, and primary data processing were performed by Personal Biotechnology Co., Ltd. (Shanghai, China). Fecal samples were extracted with pre-cooled organic solvent, sonicated at low temperature, and centrifuged, and the supernatants were used for LC-MS analysis. Chromatographic separation was performed on an Agilent 1290 Infinity ultra-high-performance liquid chromatography system, and mass spectrometric detection was carried out on a TripleTOF 6600 (SCIEX) in both positive and negative ion modes. All samples were analyzed within the same analytical batch. Raw data were converted to mzXML format using ProteoWizard and then processed with XCMS for peak extraction, alignment, and quantification. Features with a relative standard deviation (RSD) > 30% in quality control (QC) samples were removed during quality control. The remaining missing values were imputed using the k-nearest neighbors (KNN) algorithm, and signal intensities were normalized using total peak area normalization. QC-based signal correction was subsequently performed using the support vector regression (SVR) method. Because all samples were analyzed within the same analytical batch, no additional inter-batch correction was required. Positive- and negative-ion datasets were processed separately and integrated after metabolite annotation. When the same metabolite was detected in both ionization modes, the annotation with the higher Total score (identification score) was retained. Metabolite annotation was performed using the Human Metabolome Database (HMDB), MassBank, LIPID MAPS, mzCloud, and the Kyoto Encyclopedia of Genes and Genomes (KEGG). Principal component analysis (PCA) and orthogonal partial least-squares discriminant analysis (OPLS-DA) were used to evaluate overall metabolic differences, and permutation tests were used to assess model robustness. Differential metabolic features were identified based on the combined criteria of P value, fold change (FC), and variable importance in projection (VIP), with thresholds of P < 0.05 and VIP > 1, together with FC to define the direction of change. KEGG enrichment analysis was used to reveal potential biological pathways associated with differential metabolic features, and random forest analysis was used to rank representative features within this dataset.

### 2.12 Colonic transcriptome sequencing and analysis

Four biological replicates of colonic tissue from the CON, DSS, and CUR-H groups were used for transcriptome sequencing. Total RNA was extracted using TRIzol, and poly(A)+ mRNA was enriched with Oligo(dT) magnetic beads for library construction. Libraries were sequenced on an Illumina NovaSeq 6000 platform using 150-bp paired-end sequencing. Raw data were quality-controlled with fastp and aligned to the reference genome using HISAT2. Read counts were obtained with HTSeq, and fragments per kilobase of transcript per million mapped reads (FPKM) values were used for expression visualization. Differential expression analysis was performed on raw read counts using DESeq2 (22), which generated both P values and Benjamini–Hochberg-adjusted P values. Given the exploratory nature of the transcriptomic analysis and the limited number of biological replicates, genes exhibiting an absolute expression change greater than 1.5-fold and a P value < 0.05 were initially defined as exploratory differentially expressed genes (DEGs). Benjamini–Hochberg-adjusted P values were subsequently used to assess the statistical robustness of the prioritized genes. These exploratory DEGs were regarded as hypothesis-generating findings and were further evaluated through pathway-level analyses, cross-source integration, and experimental validation. Gene set enrichment analysis (GSEA) was performed on the full ranked gene list, and the normalized enrichment score (NES), P value, and false discovery rate (FDR) q value were reported (23). Genes upregulated in DSS versus CON and downregulated in CUR-H versus DSS, or showing the opposite pattern, were defined as CUR treatment-responsive genes (TRGs) and were subjected to clustering and Gene Ontology (GO) and KEGG enrichment analyses.

### 2.13 Mining of public human UC datasets, WGCNA, and network pharmacology analysis

The GSE87466 dataset (87 UC cases and 21 normal controls) was downloaded from the Gene Expression Omnibus (GEO) database. For exploratory screening, exploratory DEGs were identified using an absolute expression change greater than 1.5-fold and a P value < 0.05. For the expression matrix, the median absolute deviation (MAD) of each gene was calculated, the top 50% most variable genes were retained, and outlier samples and genes were screened using goodSamplesGenes before weighted gene co-expression network analysis (WGCNA) (24). The soft threshold was selected according to the scale-free network criterion, the minimum module size was 30, deepSplit was 3, and modules were merged at a cut height of 0.25. Genes in the modules most positively and negatively associated with disease status were intersected with the exploratory DEGs and defined as human transcriptome-supported UC-related genes. UC-related targets were retrieved from GeneCards and DrugBank, with GeneCards entries filtered above the median relevance score. These database-derived UC-related targets were combined with the human transcriptome-supported UC-related genes, standardized, and deduplicated to construct a comprehensive UC disease-associated target set (25, 26). In parallel, CUR-associated targets predicted by SwissTargetPrediction or curated in the Herbal Ingredients’ Targets (HIT) and Comparative Toxicogenomics Database (CTD) databases were combined to construct a potential CUR target set. The intersection between the comprehensive UC disease-associated target set and the potential CUR target set was defined as the candidate therapeutic target set of CUR in UC. DAVID was used for exploratory KEGG enrichment, STRING for the protein–protein interaction (PPI) network, and Cytoscape for visualization (27–29).

### 2.14 Cross-species and multi-source data integration for core candidate target identification

To clearly define the rules used to integrate evidence across species and data sources, a predefined stepwise screening strategy was applied. First, differential expression analyses were performed for DSS versus CON and CUR versus DSS. Genes that met the predefined differential expression criteria in both comparisons and exhibited opposite directions of FC were defined as TRGs. These mouse TRGs were subsequently mapped to their human orthologs. Only successfully mapped genes were retained, after which gene symbols were standardized and duplicates were removed to generate the RNA-seq-derived treatment-responsive gene set (RNA-TRG). For the human UC data, the intersection between differentially expressed genes in GSE87466 and genes within disease-associated WGCNA modules was used to construct a human transcriptome-supported UC-related gene set. The union of this gene set and UC-related targets retrieved from GeneCards and DrugBank was then taken, followed by gene-symbol standardization and deduplication, to construct a comprehensive UC disease target set. In parallel, CUR targets predicted by SwissTargetPrediction or curated in HIT and CTD were integrated to generate a potential CUR target set. The intersection between the comprehensive UC disease target set and the potential CUR target set was defined as the network pharmacology-derived drug–disease intersection target set (NP-DDI). Finally, RNA-TRG was intersected with NP-DDI, and the resulting genes were defined as cross-species, multi-source core candidate targets supported by evidence of treatment responsiveness in mice, relevance to human UC, and potential association with CUR. A PPI network was subsequently constructed for these targets, and nodes were ranked in descending order of degree centrality to prioritize candidates for subsequent experimental validation.

### 2.15 Molecular docking

Molecular docking of CUR with human SPP1 and CD44 was performed using AutoDock Vina (version 1.1.2) (30). The full-length structure of human SPP1 was obtained from the AlphaFold Protein Structure Database (AF-P10451-F1), whereas the high-resolution structure of the extracellular hyaluronan-binding domain of human CD44 was retrieved from the Protein Data Bank (PDB ID: 4PZ3) (31, 32). The three-dimensional structure of CUR was downloaded from PubChem. The receptor and ligand structures were subjected to standard preparation procedures, and molecular docking was performed using the default AutoDock Vina search parameters. For each receptor, the docking pose with the lowest (most negative) Vina score was selected and visualized using PyMOL.

### 2.16 Statistical analysis

Statistical analyses were performed using GraphPad Prism 9 and R 4.3.3. Data distribution was assessed using residual diagnostics and, where appropriate, the Shapiro–Wilk test. Normally distributed data are presented as the mean ± standard deviation (SD). Comparisons between two groups were performed using an unpaired two-tailed t test, whereas comparisons among multiple groups were performed using one-way analysis of variance (ANOVA) followed by Tukey’s multiple-comparisons test. Longitudinal repeated-measures data, including body weight and DAI, were analyzed using two-way repeated-measures ANOVA. When parametric assumptions were not met, data are presented as the median (interquartile range) and were analyzed using the Mann–Whitney U test or Kruskal–Wallis test followed by Dunn’s multiple-comparisons test. All statistical tests were two-sided, and P < 0.05 was considered statistically significant. Statistical methods and significance criteria specific to the omics analyses are described in the corresponding Methods sections.

## 3 Results

### 3.1 CUR alleviates DSS-induced disease phenotypes in UC mice

**Figure 1:**
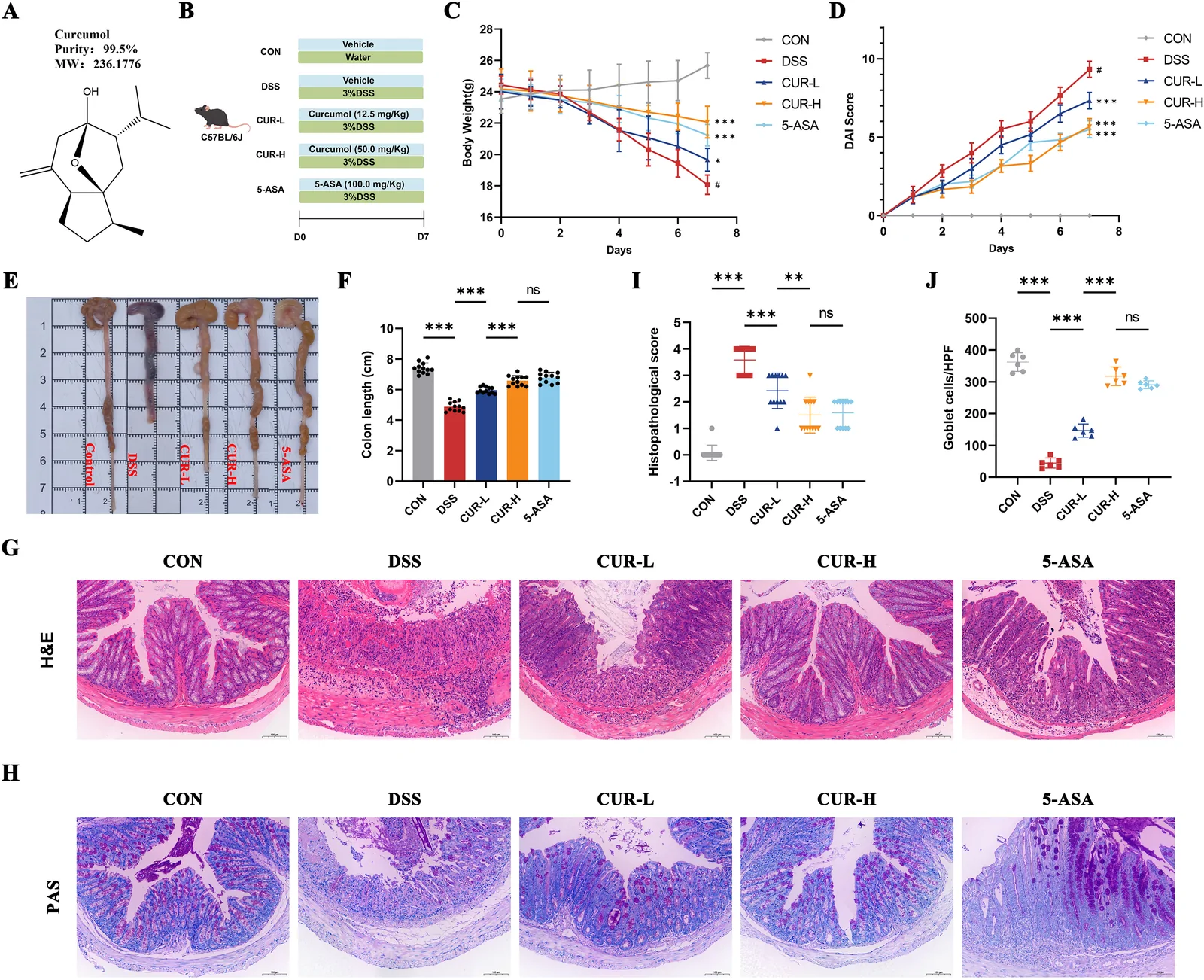
CUR alleviates DSS-induced phenotypes in the UC mouse model. **(A)** Molecular structure of CUR; **(B)** concurrent DSS-exposure/intervention design; **(C)** body weight change curve; **(D)** DAI score; **(E)** representative images of colon length; **(F)** quantitative analysis of colon length; **(G, I)** representative H&E staining and histological score; **(H, J)** representative PAS staining and goblet-cell count. #P < 0.05 versus CON; *P < 0.05, **P < 0.01, ***P < 0.001 versus DSS. Statistical comparisons in panels **(F, I, J)** are indicated by the horizontal lines in the figure. DAI, disease activity index; H&E, hematoxylin and eosin; PAS, periodic acid–Schiff; HPF, high-power field.

The chemical structure and basic compound information of CUR, including its purity (99.5%) and molecular weight (236.35), together with the overall experimental design, are shown in **Figures 1A, B**. Compared with the CON group, DSS-treated mice exhibited progressive body-weight loss, increased DAI scores, and marked colon shortening (**Figures 1C–F**). Both CUR and 5-ASA attenuated these disease manifestations to varying degrees. Compared with CUR-L, CUR-H showed more favorable numerical trends in reducing body-weight loss and DAI scores, while colon length was significantly greater in the CUR-H group than in the CUR-L group (P < 0.05; **Figure 1F**). H&E staining revealed pronounced epithelial disruption, crypt architectural distortion, and inflammatory-cell infiltration in the colonic mucosa of DSS-treated mice, all of which were alleviated following CUR administration (**Figure 1G**). Histopathological scoring further showed that both CUR-L and CUR-H attenuated DSS-induced colonic injury. The histopathological score was significantly lower in the CUR-H group than in the CUR-L group (P < 0.05), whereas no significant difference was observed between the CUR-H and 5-ASA groups (**Figure 1I**). PAS staining showed a marked loss of goblet cells following DSS exposure, whereas CUR treatment partially restored goblet-cell abundance (**Figure 1H**). Quantitative analysis further showed that goblet-cell numbers were significantly higher in the CUR-H group than in the CUR-L group (P < 0.05), whereas no significant difference was observed between the CUR-H and 5-ASA groups (**Figure 1J**). Under the 7-day experimental protocol, serum ALT, AST, creatinine, and urea levels did not differ significantly among the CON, DSS, and CUR-H groups (**Supplementary Figure 1**). Considering the more favorable overall phenotypic efficacy of CUR-H, particularly its significant improvements in colon length, histopathological score, and goblet-cell abundance compared with CUR-L, the CUR-H group was selected for subsequent exploratory multi-omics analyses.

### 3.2 CUR suppresses systemic and local colonic inflammatory responses

**Figure 2:**
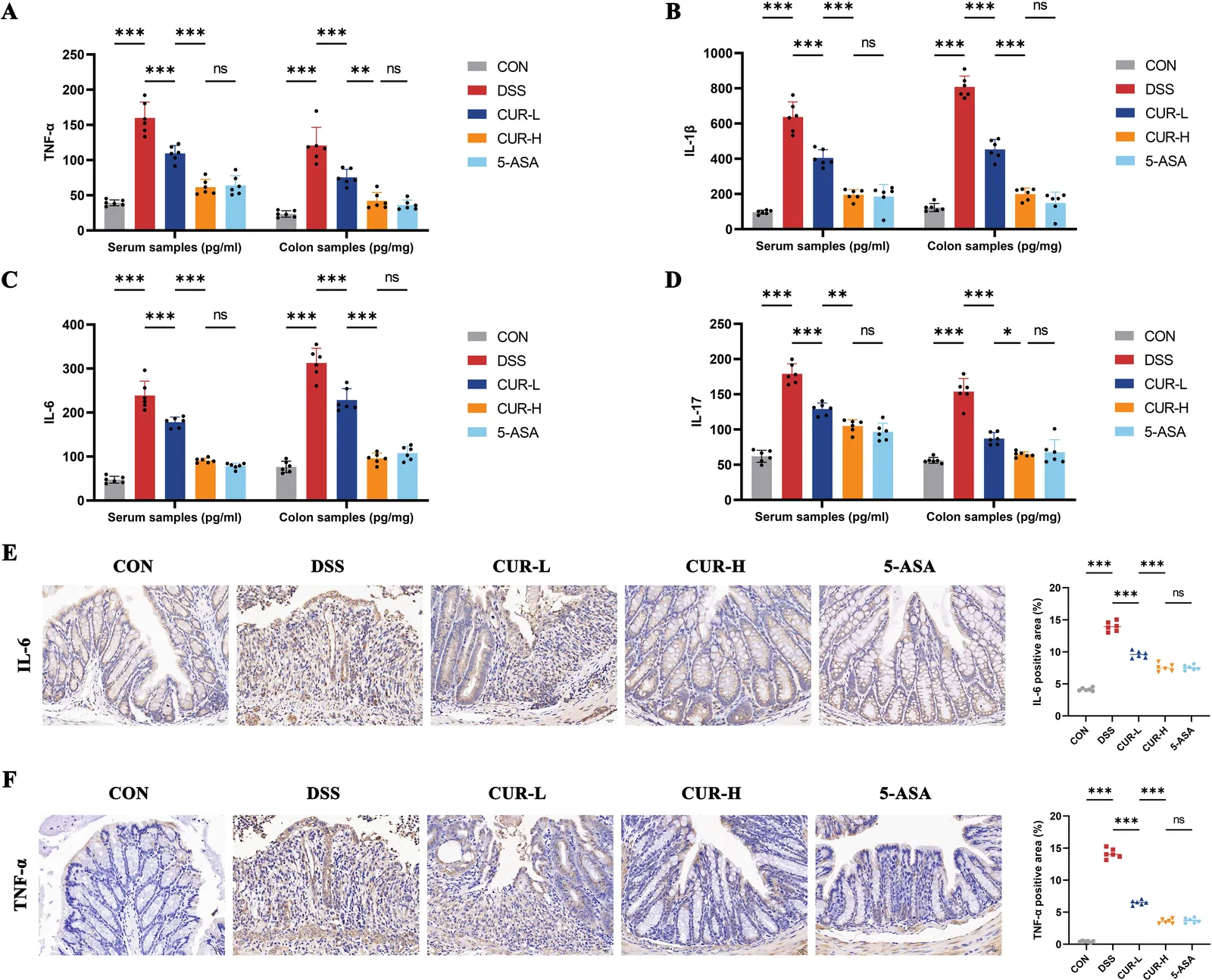
CUR suppresses DSS-induced systemic and local colonic inflammatory responses. **(A–D)** ELISA results for TNF-α, IL-1β, IL-6, and IL-17 in serum and colonic tissues; **(E, F)** representative IHC images of IL-6 and TNF-α and quantitative analysis of positive areas. *P < 0.05, **P < 0.01, ***P < 0.001. ELISA, enzyme-linked immunosorbent assay; IHC, immunohistochemistry.

ELISA results showed that, compared with the CON group, DSS treatment significantly increased TNF-α, IL-1β, IL-6, and IL-17 levels in both serum and colonic tissues, indicating pronounced systemic and local inflammatory activation in the model group (**Figures 2A–D**). Both 5-ASA and CUR reduced these pro-inflammatory cytokines to varying degrees, and CUR-H produced significantly greater reductions than CUR-L in TNF-α, IL-1β, IL-6, and IL-17 in both serum and colonic tissues (all P < 0.05; **Figures 2A–D**). IHC further showed that the positive areas of IL-6 and TNF-α in colonic tissue were markedly increased in the DSS group, whereas both were clearly reduced after CUR intervention. Quantitative analysis showed that both CUR-L and CUR-H decreased the positive areas of IL-6 and TNF-α, and these decreases were greater in the CUR-H group than in the CUR-L group (P < 0.05), with no statistically significant difference versus the 5-ASA group (**Figures 2E, F**). These results indicate that CUR concomitantly attenuates DSS-induced systemic inflammation and local colonic inflammatory activation.

### 3.3 CUR improves the expression of intestinal barrier-related molecules

**Figure 3:**
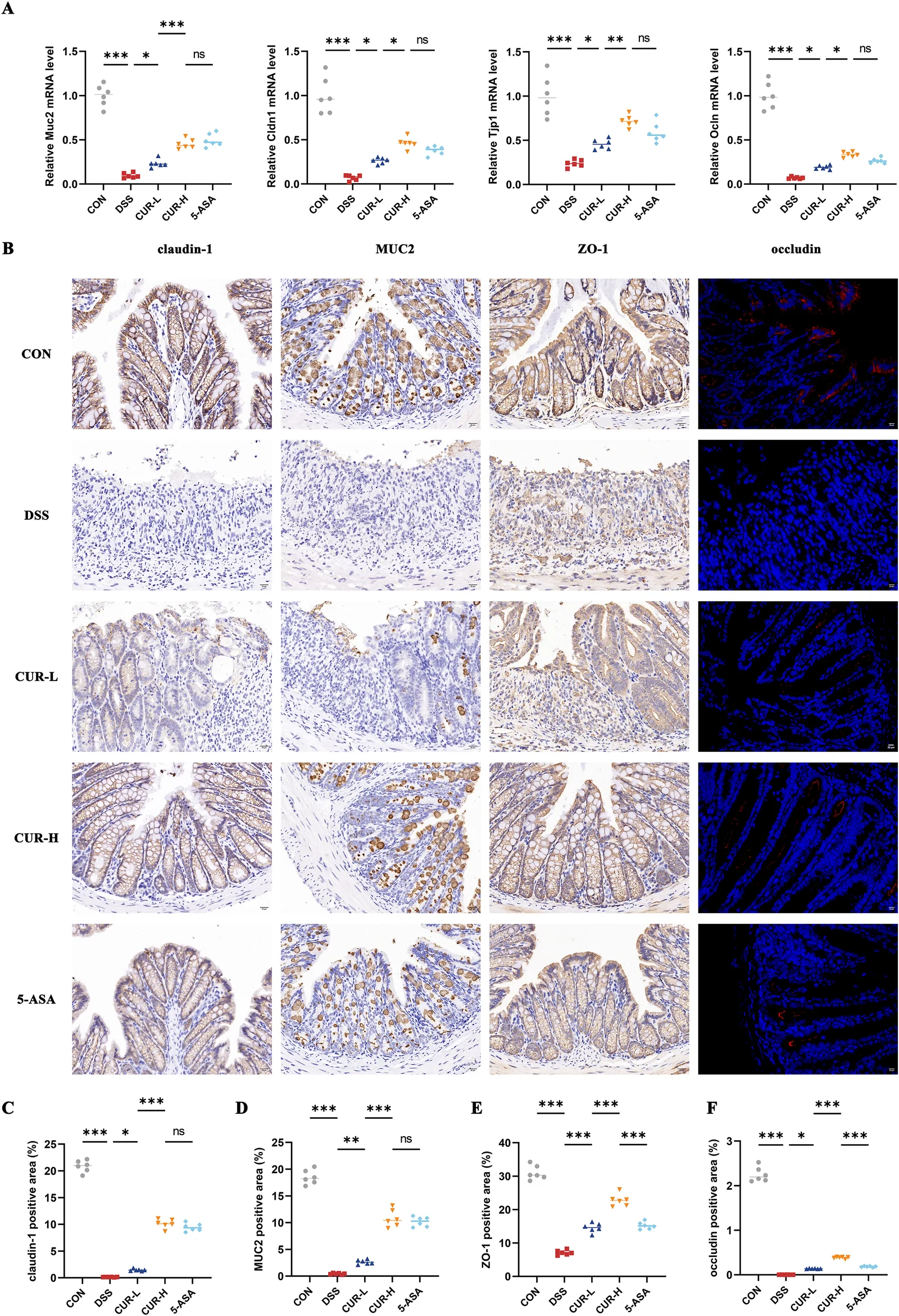
CUR improves DSS-induced expression of intestinal barrier-related molecules. **(A)** RT-qPCR results for *Cldn1*, *Muc2*, *Tjp1*, and *Ocln* in colonic tissue; **(B)** representative IHC images of claudin-1, MUC2, and ZO-1 and representative IF images of occludin; **(C–F)** positive-area quantification for claudin-1, MUC2, ZO-1, and occludin. *P < 0.05, **P < 0.01, ***P < 0.001. IF, immunofluorescence; IHC, immunohistochemistry; RT-qPCR, reverse transcription quantitative polymerase chain reaction.

RT-qPCR showed that DSS treatment significantly downregulated the mRNA expression levels of *Muc2*, *Cldn1*, *Tjp1*, and *Ocln*, whereas CUR intervention markedly restored their expression, with greater restoration in the CUR-H group than in the CUR-L group for all four markers (all P < 0.05; **Figure 3A**). IHC and IF further showed that the positive signals of MUC2, claudin-1, ZO-1, and occludin were markedly weakened in the DSS group, whereas the positive areas of these molecules were clearly increased after CUR treatment (**Figures 3B–F**). Among these indicators, CUR-H produced significantly greater recovery than CUR-L for MUC2, claudin-1, ZO-1, and occludin (all P < 0.05); recovery of claudin-1 and MUC2 was comparable to that in the 5-ASA group, whereas recovery of ZO-1 and occludin exceeded that of 5-ASA (**Figures 3B–F**).

### 3.4 CUR partially restores DSS-induced disturbances in gut microbial diversity and community structure

**Figure 4:**
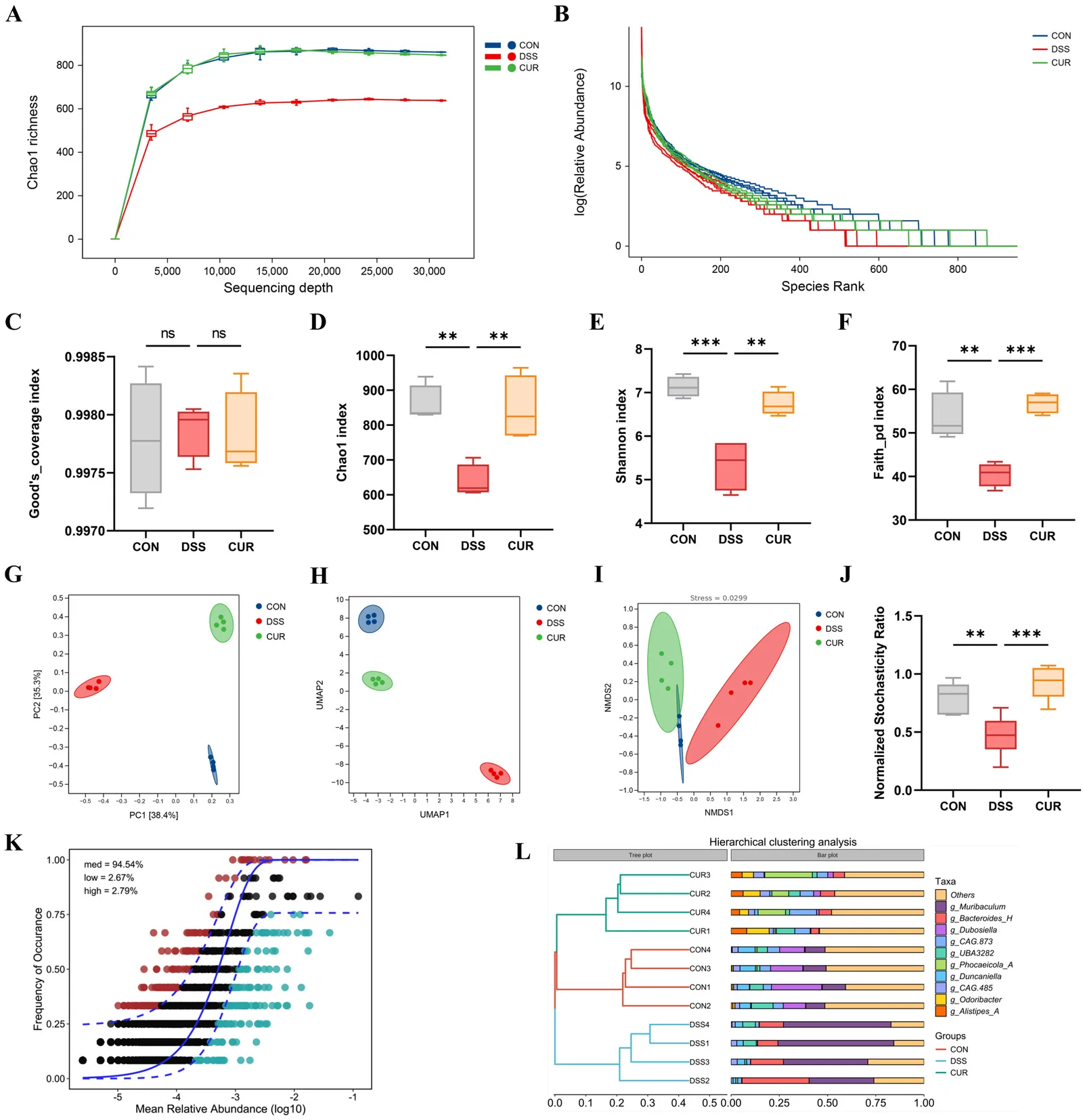
CUR partially restores DSS-induced disturbances in gut microbial diversity and community structure. **(A)** Chao1 rarefaction curves; **(B)** rank-abundance curves; **(C–F)** Good’s coverage, Chao1, Shannon, and Faith’s PD indices; **(G–I)** PCoA, UMAP, and NMDS analyses; **(J)** NST analysis; **(K)** neutral community model analysis; **(L)** hierarchical clustering heatmap at the genus level. **P < 0.01, ***P < 0.001. NMDS, non-metric multidimensional scaling; NST, normalized stochasticity ratio; PCoA, principal coordinates analysis; UMAP, uniform manifold approximation and projection. In this figure, CUR denotes the high-dose CUR group (CUR-H, 50 mg/kg/day).

The 16S rRNA sequencing rarefaction curves gradually reached a plateau with increasing sequencing depth, indicating that the current sequencing depth was sufficient to cover the major microbial information in the samples (**Figure 4A**). Rank-abundance curves showed a shorter span and a steeper slope in the DSS group, suggesting reductions in both community richness and evenness (**Figure 4B**). Further α-diversity analysis showed no statistically significant difference in Good’s coverage among the three groups (**Figure 4C**). Compared with the CON group, DSS significantly reduced the Chao1, Shannon, and Faith’s PD indices, whereas CUR intervention markedly increased these indices, indicating that CUR could partially restore the DSS-induced loss of microbial richness and diversity (**Figures 4D–F**). β-diversity analyses further supported this result. PCoA, UMAP, and NMDS all showed clear separation among the CON, DSS, and CUR groups, with the CUR group separated from the DSS group and displaying an overall shift toward the CON group, suggesting that CUR could partially correct DSS-induced disturbances in community structure (**Figures 4G–I**). NST analysis showed that the normalized stochasticity ratio decreased in the DSS group but increased markedly after CUR intervention (**Figure 4J**). Meanwhile, the neutral community model showed that most microbial members fell within the model-predicted range (**Figure 4K**), indicating an important contribution of stochastic processes to community assembly; DSS induction may have enhanced deterministic selection, whereas CUR partly restored the stochastic characteristics of community assembly. Hierarchical clustering at the genus level also showed that CUR samples clustered separately from DSS samples and shifted overall toward the CON group (**Figure 4L**).

### 3.5 CUR reshapes gut microbiota composition and is accompanied by changes in predicted functional profiles

**Figure 5:**
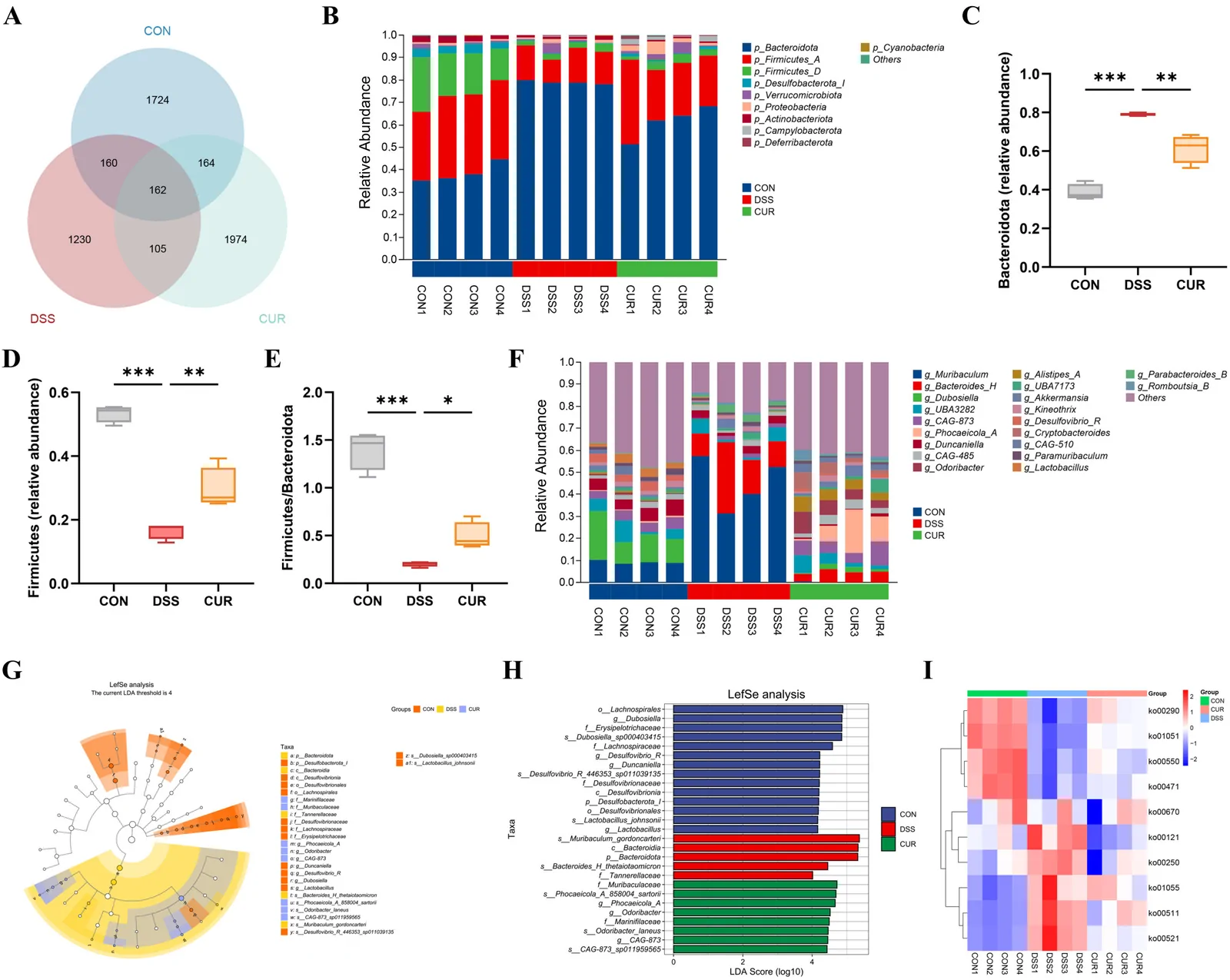
CUR reshapes gut microbiota composition and influences predicted functional profiles. **(A)** Venn diagram of shared and specific ASVs among the three groups; **(B)** bar plot of microbial composition at the phylum level; **(C–E)** relative abundance of Bacteroidota and Firmicutes and the Firmicutes/Bacteroidota ratio; **(F)** bar plot of microbial composition at the genus level; **(G)** LEfSe cladogram; **(H)** LEfSe LDA score plot (LDA > 4); **(I)** heatmap of KEGG functional profiles predicted by PICRUSt2. *P < 0.05, **P < 0.01, ***P < 0.001. ASV, amplicon sequence variant; LDA, linear discriminant analysis; LEfSe, linear discriminant analysis effect size. In this figure, CUR denotes the high-dose CUR group (CUR-H, 50 mg/kg/day).

The Venn diagram showed that the three groups shared some ASVs while also possessing group-specific ASVs, indicating that both DSS treatment and CUR intervention markedly altered microbial composition (**Figure 5A**). At the phylum level, DSS significantly increased the relative abundance of Bacteroidota and decreased that of Firmicutes, resulting in a marked reduction in the Firmicutes/Bacteroidota (F/B) ratio; these changes were partially reversed after CUR intervention (**Figures 5B–E**). The genus-level stacked bar plots further showed that the dominant microbial structure in the CUR group differed markedly from that of the DSS group and shifted overall toward the CON group (**Figure 5F**). LEfSe analysis showed enrichment of taxa such as Lachnospirales, Dubosiella, and Lactobacillus in the CON group; Bacteroidota/Bacteroidia, Tannerellaceae, and Muribaculum gordoncarteri-related taxa in the DSS group; and Muribaculaceae, Phocaeicola_A, Odoribacter, and Marinifilaceae in the CUR group (**Figures 5G, H**). PICRUSt2 functional prediction suggested that CUR-associated microbial changes were mainly related to amino acid metabolism, glycan biosynthesis and metabolism, lipid metabolism, and terpenoid/polyketide metabolism pathways (**Figure 5I**).

### 3.6 CUR partially reverses DSS-induced fecal metabolic disturbances in UC mice

**Figure 6:**
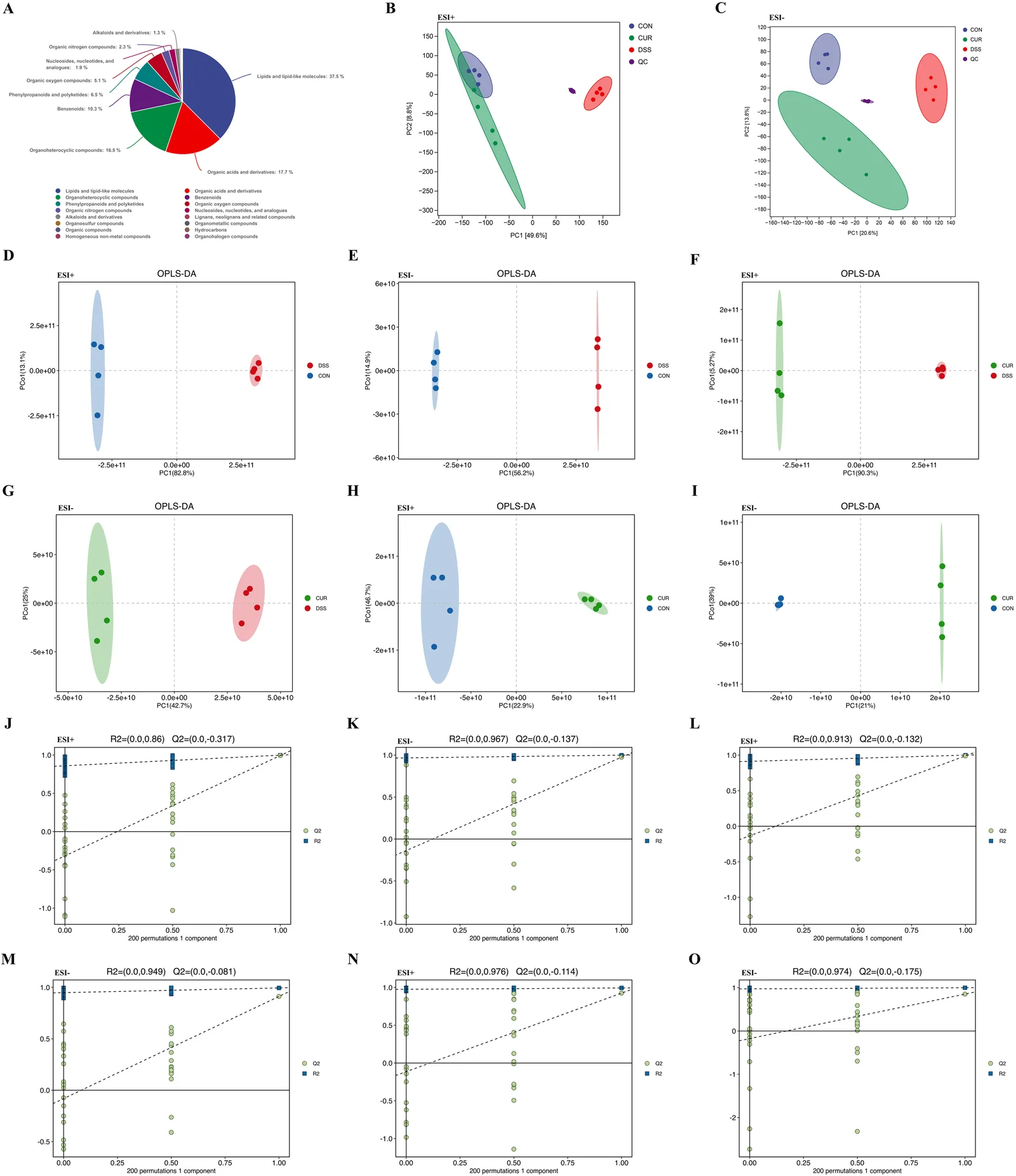
Overall characterization of fecal untargeted metabolomics. **(A)** Primary classification composition of annotated metabolic features; **(B, C)** PCA score plots in positive and negative ion modes; **(D–I)** OPLS-DA score plots for pairwise group comparisons in positive and negative ion modes, where D and E represent DSS versus CON, F and G represent CUR versus DSS, and H and I represent CUR versus CON; **(J–O)** corresponding 200-permutation tests. OPLS-DA, orthogonal partial least-squares discriminant analysis; PCA, principal component analysis; QC, quality control; ESI+, positive electrospray ionization mode; ESI−, negative electrospray ionization mode. In this figure, CUR denotes the high-dose CUR group (CUR-H, 50 mg/kg/day).

**Figure 7:**
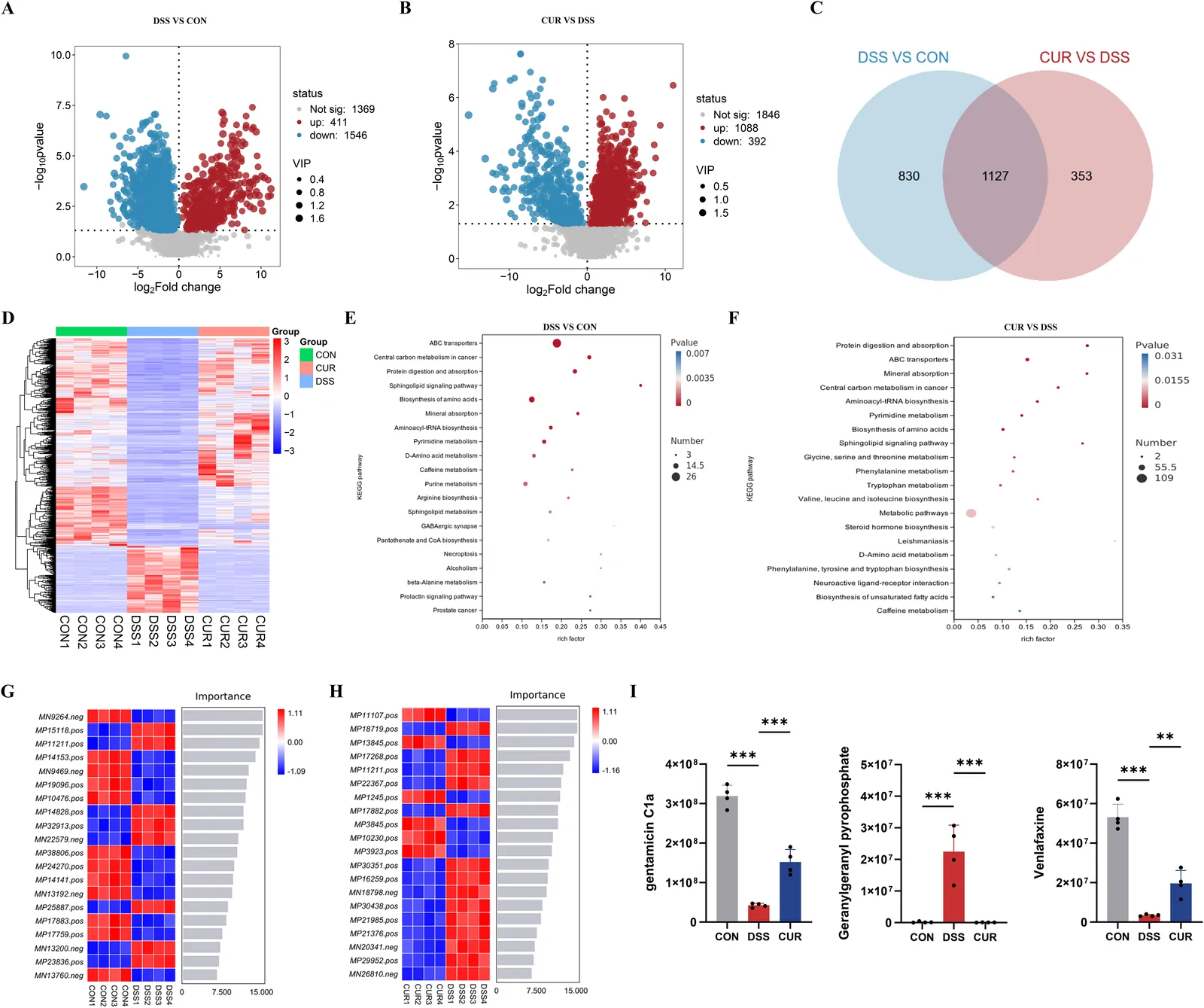
CUR partially reverses DSS-induced metabolic disturbances. **(A, B)** Volcano plots of exploratory differential metabolic features for DSS versus CON and CUR versus DSS; **(C)** Venn diagram of differential metabolic features showing opposite change trends between the two comparisons; **(D)** clustering heatmap of differential metabolic features with opposite trends; **(E, F)** KEGG enrichment analyses of differential metabolic features for DSS versus CON and CUR versus DSS; **(G, H)** top 20 representative metabolic features identified by random forest; **(I)** relative abundance changes of selected representative differential metabolic features among the three groups. **P < 0.01, ***P < 0.001. VIP, variable importance in projection. In this figure, CUR denotes the high-dose CUR group (CUR-H, 50 mg/kg/day).

A total of 3326 metabolic features were annotated in the untargeted metabolomics analysis, among which lipids and lipid-like molecules accounted for the largest proportion, followed by organic acids and derivatives, organic heterocyclic compounds, benzenoids, and phenylpropanoids/polyketides (**Figure 6A**). PCA showed tight clustering of QC samples in both positive and negative ion modes, indicating stable data quality. Meanwhile, the CON, DSS, and CUR groups were clearly separated overall, suggesting that both DSS treatment and CUR intervention significantly altered fecal metabolic features (**Figures 6B, C**). Further pairwise OPLS-DA showed clear discrimination for DSS vs CON, CUR vs DSS, and CUR vs CON in both positive and negative ion modes (**Figures 6D–I**). The corresponding permutation tests yielded Q2 intercepts below 0, indicating stable models without obvious overfitting (**Figures 6J–O**). Differential feature analysis identified 1,957 significantly altered metabolic features in DSS vs CON, including 411 upregulated and 1,546 downregulated features, and 1,480 significantly altered features in CUR vs DSS, including 1,088 upregulated and 392 downregulated features (**Figures 7A, B**). Among them, 1,127 overlapping differential metabolic features showed opposite change trends in DSS vs CON and CUR vs DSS, suggesting that CUR could reverse DSS-induced metabolic disturbances to a considerable extent (**Figures 7C, D**). KEGG enrichment analysis showed that these differential features were mainly enriched in protein digestion and absorption, ABC transporters, mineral absorption, central carbon metabolism, aminoacyl-tRNA biosynthesis, and several amino acid metabolism-related pathways (**Figures 7E, F**). Random forest analysis further screened a set of representative metabolic features with high discriminatory power, and the relative abundance changes of several representative features further supported the reversing effect of CUR on DSS-induced metabolic abnormalities (**Figures 7G–I**).

### 3.7 Colonic transcriptomics indicates that CUR reverses DSS-induced transcriptional imbalance

**Figure 8:**
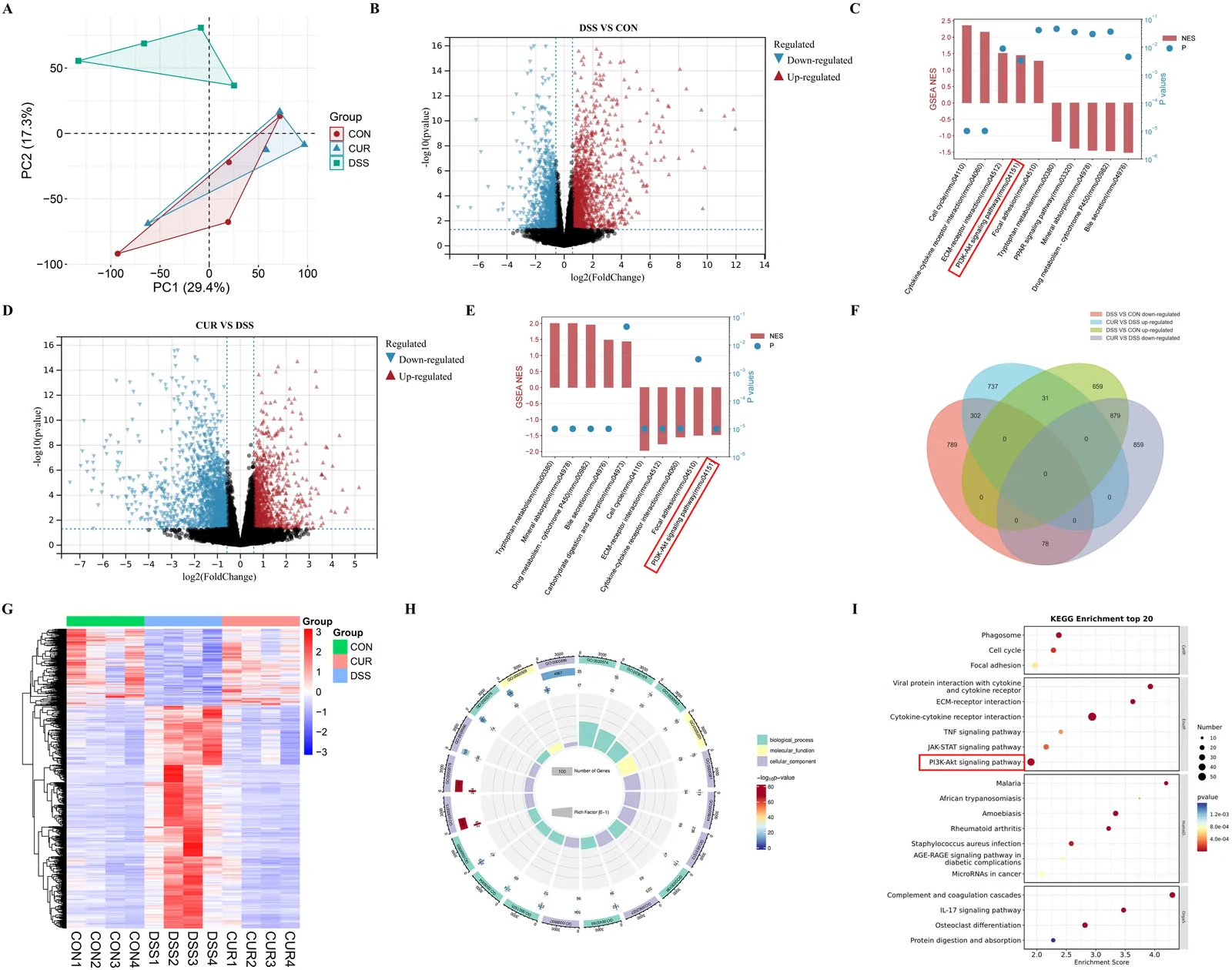
Mouse colonic transcriptomic analysis indicates that CUR partially reverses DSS-induced transcriptional imbalance. **(A)** PCA score plot; **(B)** volcano plot of differential genes in DSS versus CON; **(C)** GSEA results for DSS versus CON; **(D)** volcano plot of differential genes in CUR versus DSS; **(E)** GSEA results for CUR versus DSS; **(F)** intersection analysis of CUR treatment-responsive genes (TRGs); **(G)** clustering heatmap of TRGs; **(H)** GO enrichment analysis; **(I)** KEGG enrichment analysis. GO, Gene Ontology; GSEA, gene set enrichment analysis; NES, normalized enrichment score; PCA, principal component analysis. In this figure, CUR denotes the high-dose CUR group (CUR-H, 50 mg/kg/day).

PCA showed distinct overall transcriptomic profiles among the CON, DSS, and CUR groups. DSS samples were clearly separated from the CON and CUR samples, whereas the CUR group exhibited an overall shift toward the CON group (**Figure 8A**). Using the predefined exploratory differential-expression criteria, 2,738 DEGs were identified in DSS versus CON, including 1,569 upregulated and 1,169 downregulated genes. In CUR versus DSS, 2,886 DEGs were identified, including 1,070 upregulated and 1,816 downregulated genes (**Figures 8B, D**). GSEA showed that ECM–receptor interaction (NES = 1.507, P = 0.009, FDR q = 0.044), PI3K/Akt signaling (NES = 1.447, P = 0.003, FDR q = 0.066), and focal adhesion (NES = 1.276, P = 0.041, FDR q = 0.196) were positively enriched in DSS versus CON. In CUR versus DSS, the same pathways were negatively enriched, with NES values of −1.765 (P < 0.001, FDR q = 0.009), −1.474 (P < 0.001, FDR q = 0.117), and −1.496 (P = 0.003, FDR q = 0.102), respectively (**Figures 8C, E**; **Supplementary Tables 4**, **5**), suggesting that CUR could reverse DSS-induced inflammatory responses and extracellular matrix-related abnormalities at the transcriptional level. Among these pathways, PI3K/Akt signaling was identified as a key candidate pathway that was aberrantly enriched after DSS induction and reversed following CUR intervention. Further intersection analysis identified 1,181 CUR TRGs, including 879 genes that were upregulated in DSS versus CON and downregulated in CUR versus DSS, and 302 genes that exhibited the opposite directional pattern (**Figure 8F**). Hierarchical clustering of these TRGs showed that the expression pattern of the CUR group shifted away from that of the DSS group and toward that of the CON group (**Figure 8G**). GO and KEGG enrichment analyses of the TRGs indicated that these genes were mainly associated with inflammatory responses, cytokine activity, extracellular-matrix organization, and pathways including PI3K/Akt signaling, cytokine–cytokine receptor interaction, ECM–receptor interaction, JAK–STAT signaling, TNF signaling, and IL-17 signaling (**Figures 8H, I**).

### 3.8 Public human UC dataset analysis and network pharmacology identify candidate targets of CUR against UC

**Figure 9:**
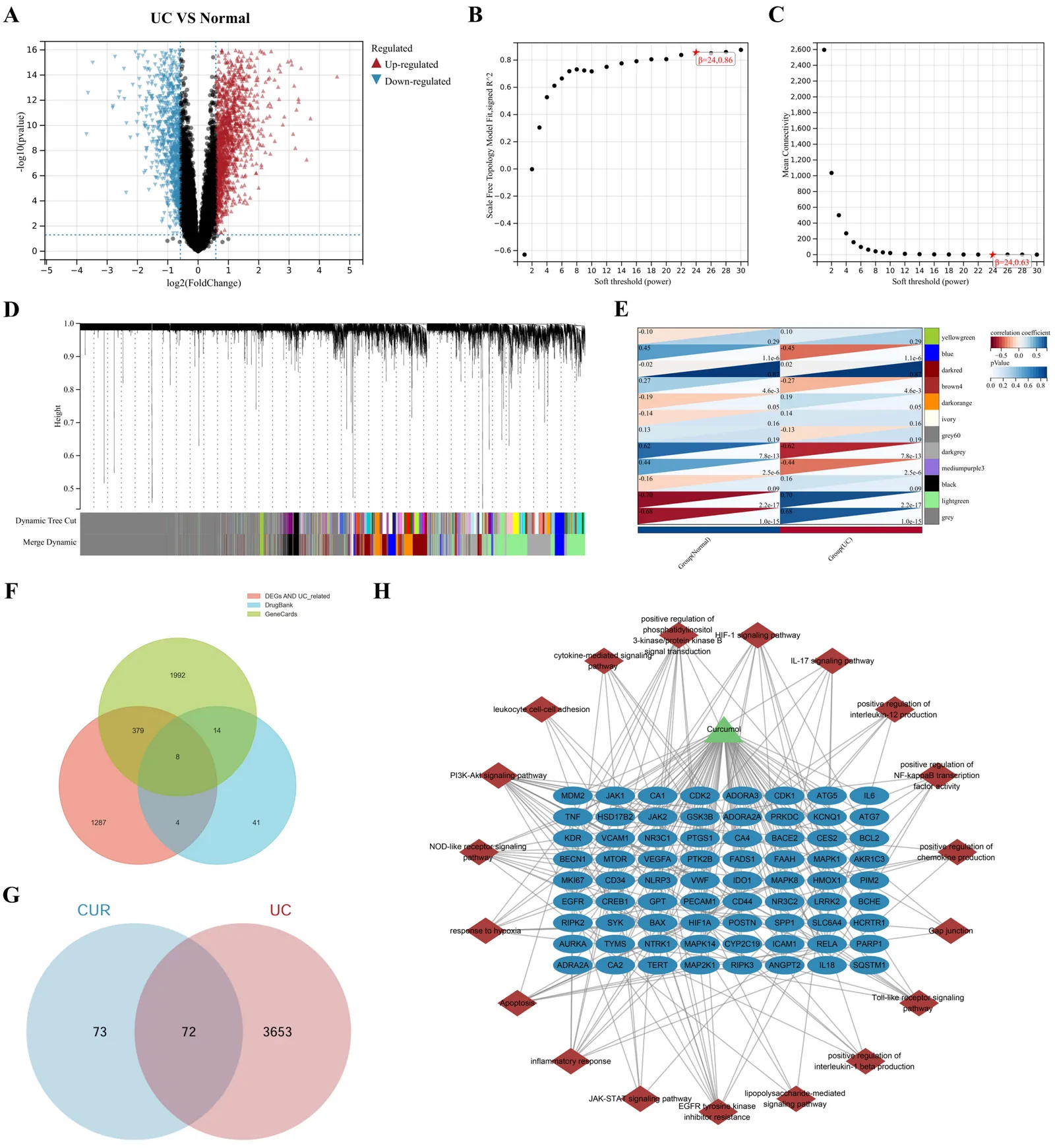
Integrative analysis of public human UC data and network pharmacology. **(A)** Exploratory differential-expression analysis of GSE87466; **(B, C)** WGCNA soft-threshold selection and scale-free network evaluation; **(D)** gene clustering tree and module assignment; **(E)** heatmap of module–phenotype correlations; **(F)** integration of the UC disease target library; **(G)** Venn diagram of potential CUR targets and UC disease targets; **(H)** CUR–target–pathway network. UC, ulcerative colitis; WGCNA, weighted gene co-expression network analysis.

In the human UC dataset, exploratory differential-expression analysis of GSE87466 yielded 2,479 differentially expressed genes (**Figure 9A**). WGCNA constructed a co-expression network based on the filtered expression matrix and identified 12 co-expression modules. Soft-threshold selection and scale-free network evaluation indicated stable network construction (**Figures 9B–D**), and module–phenotype correlation analysis further identified key modules significantly positively or negatively associated with UC status (**Figure 9E**). Genes from these disease-related modules were intersected with the exploratory differentially expressed genes and then merged with UC-related genes retrieved from GeneCards and DrugBank to build the UC disease target library (**Figure 9F**). In parallel, integration of SwissTargetPrediction-predicted and HIT/CTD-curated targets yielded a total of 145 potential CUR targets. Intersecting the CUR target set with the UC disease target library yielded 72 candidate targets of CUR against UC (**Figure 9G**). Construction of the drug–target–pathway/biological process network further suggested that CUR may coordinately intervene in UC-related pathological processes through multiple targets and pathways (**Figure 9H**). Functional enrichment of these targets mainly involved inflammatory response, cytokine-mediated signaling, PI3K/Akt, IL-17, JAK–STAT, Toll-like receptor, and NOD-like receptor signaling pathways, as well as biological processes such as response to hypoxia, apoptosis, and regulation of NF-κB transcriptional activity.

### 3.9 Integrative analysis and experimental validation suggest that SPP1/CD44/PI3K/Akt-related signaling may participate in the protective effects of CUR

**Figure 10:**
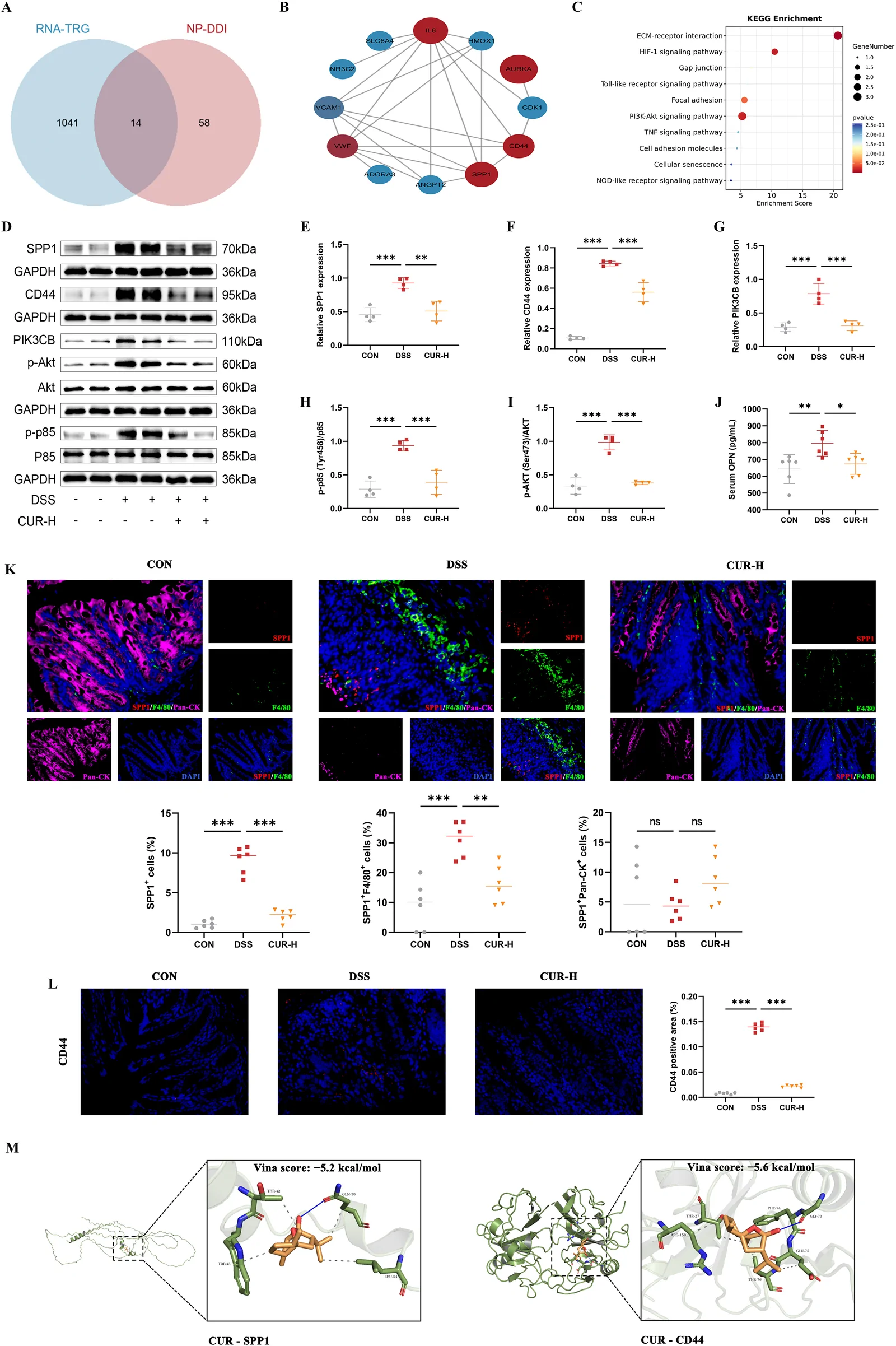
Integrative analysis of mouse colonic transcriptomics and network pharmacology, and validation of key proteins. **(A)** Intersection of mouse CUR treatment-responsive genes (TRGs) mapped to human orthologs and standardized as RNA-TRG and the network-pharmacology-derived drug–disease intersection set (NP-DDI). **(B)** STRING PPI network of 12 connected candidates; node size and color represent degree centrality. **(C)** Exploratory KEGG enrichment of the 14 candidates. **(D)** Representative Western blots of SPP1, CD44, PIK3CB, phospho-p85, total p85, phospho-Akt, total Akt, and GAPDH. **(E–G)** SPP1, CD44, and PIK3CB relative to GAPDH. **(H, I)** Phospho-p85/total p85 and phospho-Akt/total Akt. **(J)** Serum OPN (SPP1) concentration. **(K)** Multiplex TSA IF of SPP1 (red), F4/80 (green), Pan-CK (magenta), and DAPI (blue), with SPP1-positive cells among all DAPI-positive cells and F4/80-positive or Pan-CK-positive fractions among SPP1-positive cells. **(L)** CD44 IF and CD44-positive area fraction. **(M)** Exploratory CUR docking poses with the SPP1 AlphaFold model (AF-P10451-F1) and CD44 hyaluronan-binding domain (PDB 4PZ3), with Vina scores of −5.2 and −5.6 kcal/mol, respectively. *P < 0.05, **P < 0.01, ***P < 0.001. DAPI, 4′,6-diamidino-2-phenylindole; IF, immunofluorescence; OPN, osteopontin; Pan-CK, pan-cytokeratin; NP-DDI, network pharmacology-derived drug–disease intersection target set; PPI, protein–protein interaction; RNA-TRG, RNA-seq-derived treatment-responsive gene set; TSA, tyramide signal amplification.

Following ortholog mapping, RNA-TRG was intersected with the network pharmacology-derived NP-DDI, yielding 14 cross-species, multi-source core candidate genes (**Figure 10A**). PPI network analysis and degree-centrality ranking showed that *IL6*, *AURKA*, *CD44*, *SPP1*, and *VWF* occupied central positions within the network (**Figure 10B**). KEGG enrichment analysis indicated that these core candidates were associated with ECM–receptor interaction, focal adhesion, HIF-1 signaling, Toll-like receptor signaling, and PI3K/Akt signaling (**Figure 10C**). Among them, SPP1 and CD44 were core candidate molecules commonly highlighted by the multi-source integrative analysis and were strongly related to the PI3K/Akt pathway. On this basis, SPP1, CD44, and the associated signaling components were further evaluated experimentally. Western blotting showed that, compared with the CON group, DSS markedly increased the protein abundance of SPP1, CD44, and PIK3CB in colonic tissues, accompanied by significant increases in the p-p85/p85 and p-Akt/Akt ratios. CUR-H intervention significantly attenuated all of these changes (**Figures 10D–I**). Serum ELISA further showed that the concentration of OPN, the protein encoded by SPP1, was significantly elevated in the DSS group and reduced following CUR-H intervention (**Figure 10J**). IF analysis showed that the proportion of SPP1-positive cells and the CD44-positive area in the colonic mucosa were markedly increased in the DSS group and significantly reduced following CUR-H intervention (**Figures 10K, L**). Multiplex IF colocalization analysis further revealed that the F4/80-positive fraction within the SPP1-positive cell population was significantly increased in the DSS group and reduced by CUR-H treatment. In contrast, no statistically significant differences in the Pan-CK-positive fraction within the SPP1-positive cell population were observed among the groups (**Figure 10K**). Molecular docking predicted that CUR could bind within putative pockets of both SPP1 and CD44, with Vina scores of −5.2 and −5.6 kcal/mol, respectively, suggesting potential interactions between CUR and these two proteins (**Figure 10M**). Taken together, the multi-source integrative analysis and experimental validation suggest that SPP1/CD44/PI3K/Akt-related signaling may contribute to the protective effects of CUR against DSS-induced UC in mice.

## 4 Discussion

By integrating pharmacodynamic and histological phenotypes with gut microbiome profiling, fecal metabolomics, mouse colonic transcriptomics, public human UC transcriptomic data, and network pharmacology, this study established a multilevel framework for interpreting CUR action in DSS-induced UC mice. CUR ameliorated disease activity and histological injury, suppressed systemic and colonic inflammation, partially restored goblet-cell abundance and barrier-related molecules, and shifted microbial, metabolic, and host transcriptional profiles toward the control state. Cross-source integration further converged on SPP1 and CD44 and implicated PI3K/Akt-related signaling, supported by protein abundance, phosphorylation, and cellular localization data. These findings connect CUR-associated phenotypic protection with coordinated changes in the mucosal barrier, intestinal microenvironment, and host signaling rather than an isolated inflammatory endpoint (12–14).

The clinical relevance of the DSS model lies in its ability to reproduce several manifestations and pathological features observed in active human UC, including weight loss, diarrhea and rectal bleeding, epithelial erosion, crypt injury, goblet-cell depletion, barrier disruption, and inflammatory-cell infiltration; colon shortening provides an additional model-specific indicator of disease severity. Its reproducibility and sensitivity to epithelial and microbial perturbations make it particularly suitable for evaluating mucosal protection. Nevertheless, the present 7-day protocol represents acute chemically induced injury: DSS initiates inflammation through direct epithelial damage and predominantly innate immune activation, whereas human UC is a chronic, relapsing, and heterogeneous disease arising from sustained interactions among genetic susceptibility, adaptive and innate immunity, environmental factors, and the gut microbiota. Moreover, because CUR was administered concurrently with DSS, the present study primarily evaluates protection during disease induction rather than treatment of established disease. Accordingly, these results support the preclinical biological plausibility of CUR but should not be directly extrapolated to clinical efficacy (33–35). Against this pathophysiological background, parallel improvement in inflammatory and barrier-related indices is more informative than cytokine suppression alone (36). CUR reduced serum and colonic TNF-α, IL-1β, IL-6, and IL-17 levels, while increasing goblet-cell abundance and partially restoring MUC2, claudin-1, ZO-1, and occludin expression. Together with attenuated histological injury, these concordant changes indicate that CUR-associated protection involved both the mucus layer and epithelial tight junctions. The combined recovery of PAS-positive goblet cells and MUC2, together with tight-junction proteins, may help interrupt the reciprocal reinforcement between barrier failure and inflammatory amplification (34, 36).

The microbiome and metabolomics findings extend this interpretation to the intestinal ecological and metabolic environment. DSS reduced microbial richness and diversity, altered community structure, and disrupted fecal metabolic profiles, whereas CUR partially shifted these features toward the control state. The principal implication is community-level remodeling rather than dependence on a single taxon or metabolite. NST and neutral community model analyses suggested stronger deterministic selection after DSS exposure and a rebound in stochastic features after CUR intervention, possibly reflecting reduced inflammation-associated ecological filtering as the mucosal environment improved. Predicted microbial functions and metabolite enrichment converged on amino acid and lipid metabolism, glycan biosynthesis, and transport-related processes, indicating that taxonomic remodeling was accompanied by broader metabolic reorganization relevant to UC (37–40).

Host-side analyses yielded a more focused mechanistic signal. Colonic transcriptomics indicated that DSS-associated enrichment of PI3K/Akt signaling, ECM–receptor interaction, and focal adhesion shifted in the opposite direction after CUR treatment. Integration with public human UC transcriptomic data and network pharmacology further prioritized SPP1 and CD44 as cross-species, multi-source candidate targets. SPP1 is a pleiotropic ligand involved in adhesion, chemotaxis, immune regulation, and tissue remodeling, whereas CD44, one of its major receptors, contributes to immune-cell migration and cell–matrix interactions (41–43). SPP1-positive macrophages have also been implicated in aberrant macrophage–fibroblast communication in inflamed UC tissue (44). Consistent with this context, CUR reduced DSS-induced increases in SPP1, CD44, and PIK3CB protein abundance and in the p-p85/p85 and p-Akt/Akt ratios. Multiplex immunofluorescence further showed that CUR reduced the F4/80-positive fraction within the SPP1-positive population, whereas the Pan-CK-positive fraction remained unchanged, suggesting that macrophage-associated SPP1 may represent a relevant component of the CUR-responsive mucosal microenvironment. Given the context-dependent role of PI3K/Akt in epithelial homeostasis and inflammatory activation, these changes are best interpreted as attenuation of disease-associated pathway overactivation rather than generalized pathway inhibition. Collectively, the convergent transcriptomic and experimental evidence supports SPP1/CD44-associated PI3K/Akt signaling as a candidate network linked to CUR-mediated protection (45–48).

These findings complement the recent report that CUR ameliorates DSS-induced UC through PXR activation and transcriptional repression of STING signaling (11). Together, the available evidence suggests that CUR may act across interconnected modules involving inflammatory sensing, barrier integrity, the intestinal microenvironment, and immune-cell communication rather than through a single isolated signaling axis.

Several limitations should be acknowledged. Each omics analysis included only four biological replicates per group, limiting the robustness of individual differential features. PICRUSt2-derived functions and untargeted metabolite annotations remain inferential and require confirmation through higher-resolution functional profiling and targeted metabolomics. Although SPP1 and CD44 were supported across mouse, human, network, protein, phosphorylation, and cellular-localization layers, their causal contributions and the directionality of the SPP1/CD44–PI3K/Akt relationship were not tested by genetic, pharmacological, or cell-specific perturbation; exploratory molecular docking likewise does not establish direct target engagement. Finally, the necessity of microbiota changes for CUR efficacy was not examined using microbiota depletion, fecal microbiota transplantation, or defined colonization. Future studies combining pathway perturbation, cell-specific validation, microbiota-transfer experiments, and targeted metabolite quantification are needed to distinguish causal mediators from secondary consequences of disease improvement.

## 5 Conclusions

In conclusion, concurrent CUR administration during DSS exposure, particularly at the higher dose, ameliorated DSS-induced UC phenotypes by reducing systemic and colonic inflammation, alleviating histopathological injury, restoring goblet-cell abundance, and recovering mucosal barrier-related molecules. Integrated microbiome, metabolomic, and transcriptomic analyses further showed that CUR shifted DSS-disrupted microbial communities, fecal metabolic profiles, and colonic transcriptional patterns toward the control state, supporting coordinated modulation of the host–microbiota–metabolism axis. Cross-species integration of mouse colonic transcriptomics, public human UC data, and network pharmacology, followed by experimental validation, further identified SPP1/CD44/PI3K/Akt-related signaling, particularly macrophage-associated SPP1, as a candidate host-side mechanism linked to the protective effects of CUR. Collectively, these findings suggest that CUR exerts multilevel protection by coordinately regulating interconnected inflammatory, mucosal barrier, and microecological–metabolic processes rather than acting through a single inflammatory target. This study broadens the mechanistic basis for natural-product intervention in UC and provides a rationale for further causal validation of the SPP1/CD44/PI3K/Akt-related signaling axis and continued evaluation of CUR as a multi-target natural bioactive candidate for UC intervention.

## Data Availability

The original contributions presented in the study are publicly available. The RNA-sequencing data generated in this study have been deposited in the NCBI Sequence Read Archive (SRA) under BioProject accession number PRJNA1516208 and can be found here: https://www.ncbi.nlm.nih.gov/sra/PRJNA1516208.
