# Supplementary material for: Integrated multi-omics, network pharmacology, and experimental validation reveal the protective mechanisms of curcumol against DSS-induced ulcerative colitis
Source: Front Immunol. 2026 Aug 28;17:1852138. doi: 10.3389/fimmu.2026.1852138 (PMC13561950; doi:10.3389/fimmu.2026.1852138)
Supplement: Supplementary file 1 [file SupplementaryFile1.pdf]

Supplementary Material

Supplementary Table S1. Information on ELISA kits

Manufacturer (city, country)	Analyte	Catalog no.
Multisciences (Lianke) Biotech Co., Ltd. (Hangzhou, China)	Mouse IL-1β	EK201B
Multisciences (Lianke) Biotech Co., Ltd. (Hangzhou, China)	Mouse IL-6	EK206
Multisciences (Lianke) Biotech Co., Ltd. (Hangzhou, China)	Mouse IL-17A	EK217EG
Multisciences (Lianke) Biotech Co., Ltd. (Hangzhou, China)	Mouse TNF-α	EK282EGA
Multisciences (Lianke) Biotech Co., Ltd. (Hangzhou, China)	Mouse OPN	EK2135

Supplementary Table S2. RT-qPCR primer sequences

Gene	Forward primer (5′ → 3′)	Reverse primer (5′ → 3′)
Gapdh	CACCCACTCCTCCACCTTTGAC	GTCCACCACCCTGTTGCTGTAG
Muc2	AGGGCTCGGAACTCCAGAAA	CCAGGGAATCGGTAGACATCG
Cldn1	AGCACCGGGCAGATACAGT	GCCAATTACCATCAAGGCTCG
Tjp1 (ZO-1)	GGGCCATCTCAACTCCTGTA	AGAAGGGCTGACGGGTAAAT
Ocln	TGAAAGTCCACCTCCTTACAGA	CCGGATAAAAAGAGTACGCTGG

Supplementary Table S3. Antibodies used for IHC, IF, multiplex TSA IF, and Western blotting

Antibody	Catalog no.	Manufacturer (city, country)	Application and dilution
PIK3CB	ab151549	Abcam (Cambridge, UK)	WB, 1:1,000
Phospho-PI3K p85	341468	ZEN-BIOSCIENCE (Chengdu, China)	WB, 1:1,000
PI3K p85α	60225-1-Ig	Proteintech Group (Wuhan, China)	WB, 1:2,000
Phospho-Akt	4060	Cell Signaling Technology (Danvers, MA, USA)	WB, 1:1,000
Akt	60203-2-Ig	Proteintech Group (Wuhan, China)	WB, 1:5,000
SPP1/OPN	22952-1-AP	Proteintech Group (Wuhan, China)	WB, 1:1,000; IF/TSA IF, 1:200
CD44	85163-5-RR	Proteintech Group (Wuhan, China)	WB, 1:1,000; IF, 1:200
IL-6	83795-7-RR	Proteintech Group (Wuhan, China)	IHC, 1:200
TNF-α	17590-1-AP	Proteintech Group (Wuhan, China)	IHC, 1:200
Claudin-1	28674-1-AP	Proteintech Group (Wuhan, China)	IHC, 1:200
ZO-1	21773-1-AP	Proteintech Group (Wuhan, China)	IHC, 1:200
MUC2	27675-1-AP	Proteintech Group (Wuhan, China)	IHC, 1:200

Antibody	Catalog no.	Manufacturer (city, country)	Application and dilution
Occludin	27260-1-AP	Proteintech Group (Wuhan, China)	IF, 1:200
F4/80	28463-1-AP	Proteintech Group (Wuhan, China)	Multiplex TSA IF, 1:200
Pan-cytokeratin (Pan-CK)	26411-1-AP	Proteintech Group (Wuhan, China)	Multiplex TSA IF, 1:200
GAPDH	10494-1-AP	Proteintech Group (Wuhan, China)	WB, 1:10,000

Supplementary Table S4. Selected KEGG gene sets from GSEA in DSS versus CON

Term	NES	P value	FDR q value
Cell cycle (mmu04110)	2.356	<0.001	<0.001
Cytokine-cytokine receptor interaction (mmu04060)	2.157	<0.001	<0.001
ECM–receptor interaction (mmu04512)	1.507	0.009	0.044
PI3K/Akt signaling pathway (mmu04151)	1.447	0.003	0.066
Focal adhesion (mmu04510)	1.276	0.041	0.196
Tryptophan metabolism (mmu00380)	-1.172	0.045	0.187
PPAR signaling pathway (mmu03320)	-1.385	0.035	0.154
Mineral absorption (mmu04978)	-1.447	0.029	0.107
Drug metabolism - cytochrome P450 (mmu00982)	-1.458	0.036	0.112
Bile secretion (mmu04976)	-1.513	0.004	0.106

Note: NES, normalized enrichment score; FDR, false discovery rate. Pathways with $P < 0.05$ and FDR $q < 0.25$ were considered significantly enriched according to the predefined GSEA criteria.

Supplementary Table S5. Selected KEGG gene sets from GSEA in CUR-H versus DSS

Term	NES	P value	FDR q value
Tryptophan metabolism (mmu00380)	2.002	<0.001	0.003
Mineral absorption (mmu04978)	2.000	<0.001	0.003
Drug metabolism - cytochrome P450 (mmu00982)	1.952	<0.001	0.003
Bile secretion (mmu04976)	1.478	<0.001	0.083
Carbohydrate digestion and absorption (mmu04973)	1.430	0.045	0.095
Cell cycle (mmu04110)	-1.965	<0.001	0.001
ECM–receptor interaction (mmu04512)	-1.765	<0.001	0.009
Cytokine-cytokine receptor interaction (mmu04060)	-1.547	<0.001	0.065
Focal adhesion (mmu04510)	-1.496	0.003	0.102
PI3K/Akt signaling pathway (mmu04151)	-1.474	<0.001	0.117

Note: NES, normalized enrichment score; FDR, false discovery rate. Pathways with $P < 0.05$ and FDR $q < 0.25$ were considered significantly enriched according to the predefined GSEA criteria.

Supplementary Table S6. Public access information for datasets generated in this study

Data category	Subfolder name within 1852138_Raw_Data
Colonic transcriptome sequencing	Colonic_Transcriptome_Sequencing
Fecal 16S rRNA sequencing	Fecal_16S_rRNA_Sequencing
Fecal untargeted metabolomics	Fecal_Untargeted_Metabolomics

Note: The raw datasets are publicly available for viewing and download via the Jianguoyun public link: https://www.jianguoyun.com/p/DWutp6gQoLOGCxj616cGIAA The link opens the main folder "1852138_Raw_Data," which contains the three subfolders listed above.

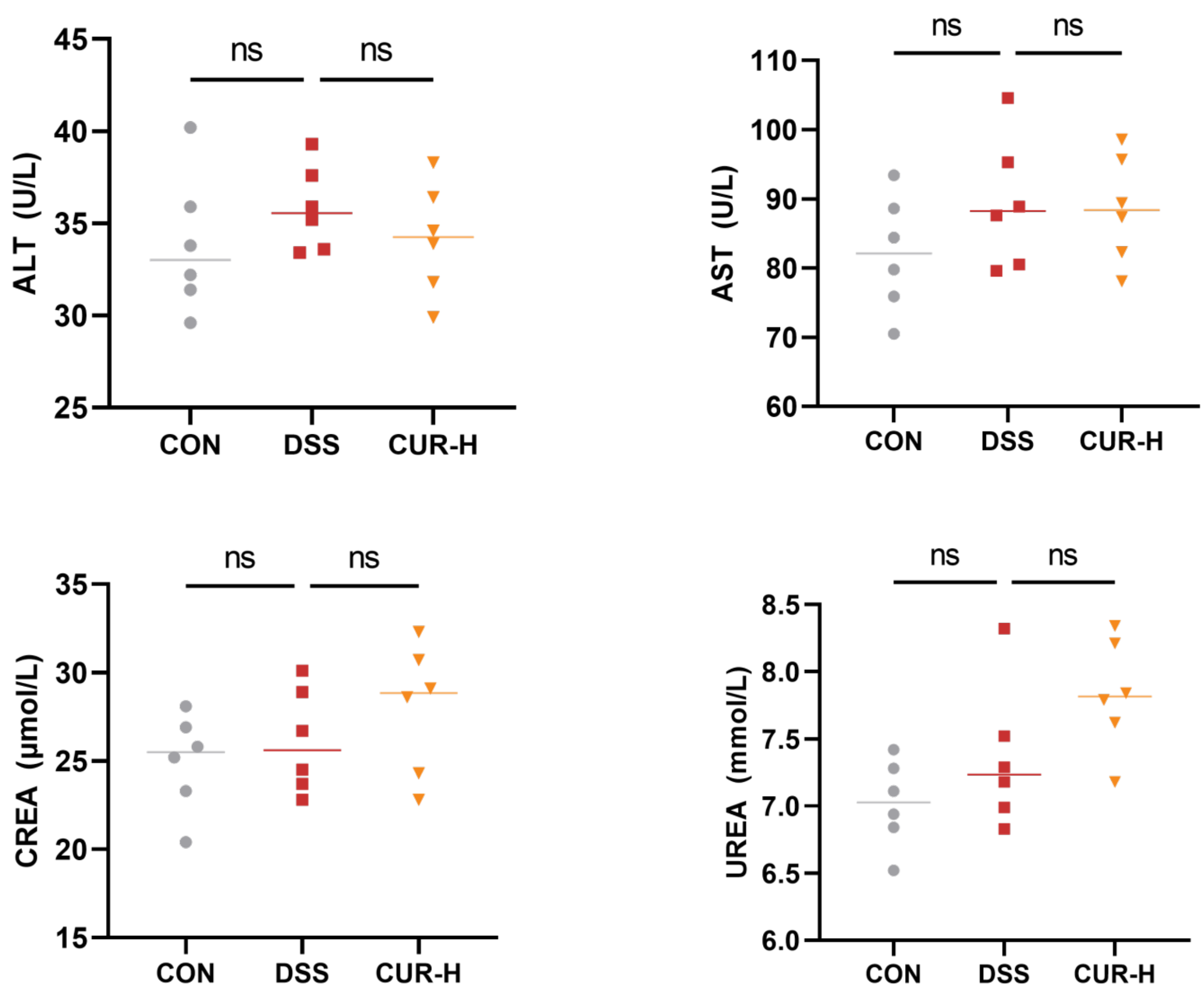

Supplementary Figure S1. Short-term serum biochemical indices in CON, DSS, and CUR-H mice

Serum alanine aminotransferase (ALT), aspartate aminotransferase (AST), creatinine (CREA), and urea (UREA) were measured after the 7-day concurrent DSS-exposure/intervention protocol. Each symbol represents one mouse (n = 6 per group), and horizontal lines indicate group means. The displayed pairwise comparisons were multiplicity-adjusted as described in Statistical Analysis. ns, not significant. These four indices provide preliminary short-term biochemical tolerability information and do not constitute a comprehensive toxicological assessment.
